# A cognitive-behavioral intervention for emotion regulation in adults with high-functioning autism spectrum disorders: study protocol for a randomized controlled trial

**DOI:** 10.1186/1745-6215-14-231

**Published:** 2013-07-23

**Authors:** Miho Kuroda, Yuki Kawakubo, Hitoshi Kuwabara, Kazuhito Yokoyama, Yukiko Kano, Yoko Kamio

**Affiliations:** 1 Department of Psychology, Shukutoku University, 200 Daiganji-machi, Chuo-ku, Chiba-shi 260-8701, Japan; 2 Department of Child Neuropsychiatry, Graduate School of Medicine, The University of Tokyo, 7-3-1, Hongo, Bunkyo-ku, Tokyo 113-8655, Japan; 3 Department of Child and Adolescence Mental Health, National Institute of Mental Health, National Center of Neurology and Psychiatry, 4-1-1 Ogawahigashicho, Kodairashi, Tokyo 187-8553, Japan; 4 Department of Epidemiology and Environmental Health, Juntendo University Faculty of Medicine, 2-1-1 Hongo, Bunkyo-ku, Tokyo 113-8421, Japan

**Keywords:** Autism spectrum disorders, Emotion regulation, High-functioning adults, Cognitive-behavioral therapy, Randomized controlled trial

## Abstract

**Background:**

Adults with high-functioning autism spectrum disorders (ASD) have difficulties in social communication; thus, these individuals have trouble understanding the mental states of others. Recent research also suggests that adults with ASD are unable to understand their own mental states, which could lead to difficulties in emotion-regulation. Some studies have reported the efficacy of cognitive-behavioral therapy (CBT) in improving emotion-regulation among children with ASD. The current study will investigate the efficacy of group-based CBT for adults with ASD.

**Methods/Design:**

The study is a randomized, waitlist controlled, single-blinded trial. The participants will be 60 adults with ASD; 30 will be assigned to a CBT group and 30 to a waitlist control group. Primary outcome measures are the 20-item Toronto Alexithymia Scale, the Coping Inventory for Stressful Situations, the Motion Picture Mind-Reading task, and an ASD questionnaire. The secondary outcome measures are the Center for Epidemiological Studies Depression Scale, the World Health Organization Quality of Life Scale 26-item version, the Global Assessment of Functioning, State-trait Anxiety Inventory, Social Phobia and Anxiety Inventory, and Liebowitz Social Anxiety Scale. All will be administered during the pre- and post-intervention, and 12 week follow-up periods. The CBT group will receive group therapy over an 8 week period (one session per week) with each session lasting approximately 100 minutes. Group therapy will consist of four or five adults with ASD and two psychologists. We will be using visual materials for this program, mainly the Cognitive Affective Training kit.

**Discussion:**

This trial will hopefully indicate the efficacy of group-based CBT for adults with high- functioning ASD.

**Trial registration:**

This trial was registered in The University Hospital Medical Information Network Clinical Trials Registry No. UMIN000006236.

## Background

Autism spectrum disorders (ASD) are a group of developmental disorders that include qualitative impairment in interpersonal communication as a core symptom. Even for an adult with high-functioning ASD, whose intellectual development is within the normal range, it is difficult to overcome difficulties in understanding the thoughts and emotions of others; this leads to impairments in interpersonal communication [1-3]. In recent years, studies have shown that an individual may not only find it difficult to recognize the emotions of others but also struggle with identifying one’s own emotions and matching the nature of those emotions with the appropriate strength and language given the current context; this can lead to difficulties in identifying or expressing their own mental states [4,5]. Some studies have shown that 50% of ASD adults have alexithymia, which is a personality construct characterized by a sub-clinical inability to identify and describe one’s own emotions [6,7]. This inability to identify or express one’s own mental states, coupled with a lack of emotion recognition, makes it even more difficult to establish mutual relationships. Failure to adapt to a group may become seriously affected and lead to interpersonal difficulties. Adults with ASD often present with one or more co-morbid disorders, such as anxiety or depression [8,9]. In many cases, a combination of mood disorders and anxiety arises due to chronic stress within a group. Hence, the treatment of patients with underlying ASD is a major issue for the mental health field. Even if symptomatic treatment is successful in relieving psychiatric symptoms, adults with ASD still find it difficult to adapt to society due to interpersonal communication difficulties. Recent research has also suggested that there are many adults with undiagnosed ASD among individuals who receive treatment for other psychiatric disorders. Many are diagnosed with ASD in adulthood without noticeable ASD symptoms during childhood [10].

Cognitive-behavioral therapy (CBT) interventions are being implemented within small group or individual therapy, with the aim of improving the regulation of emotions associated with ASD difficulties [11-15]. Sofronoff and colleagues [12] examined 71 children, aged 10 to 12 years, diagnosed with Asperger’s syndrome (AS). In some cases, the children’s parents were randomly assigned to one of three conditions (child-only intervention, child and parent intervention, and waitlist control). Small-group CBT was carried out, and the results for the three groups were compared among the three groups. Each of the intervention groups contained three participants, matched on sex and age. Two graduate student therapists conducted CBT for each group. There were 23 participants, over eight groups, in the child-only intervention group. Although there was no direct parental involvement, activities were explained after the sessions, and parents were instructed to have their children perform tasks at home. There were 25 participants, over nine groups, in the child and parent intervention condition. The interventions for the children in this condition were the same as those in the child-only intervention condition, with one psychotherapist for each group, which also included two parents. Twenty-three individuals were assigned to the control group. Six 2 hour sessions, during which participants studied how to be emotionally aware and use appropriate methods for coping with emotions, were conducted over 1 week. Results were examined via children’s self-assessments using the “James and the Maths Test”, a story describing anxiety about a math test. The parents also completed an assessment using the Spence Child Anxiety Scale and Social Worries Questionnaire–Parent. These assessments were performed at pre- and post-intervention, and also during a follow-up session (6 weeks later). A significant intervention effect was observed when both children and parents took part in the intervention; the child-only intervention was the next most effective treatment. Furthermore, a randomized comparative trial conducted by Sofronoff and colleagues [13] revealed similar results for a small-group CBT intervention to help with anger control. Based on the efficacy of the emotion-regulation which these studies showed, the Cognitive Affective Training Kit (The Cat-kit) was developed [16]. It is designed to help individuals with ASD become aware of how their thoughts, feelings and actions all interact and, in the process of using the various visual components, they share their insights with others.

White and colleagues [17] developed a manual-based CBT program to target anxiety symptoms as well as social skill deficit in adolescents with ASD. Their treatment program includes 12 to 16 individual sessions of 50 to 75 minutes with session content tailored to the individual. Small-group CBT starts approximately 3 weeks after the start of the individual sessions. The small group sessions continue over five, 60 minute sessions, during alternate weeks. Parental participation during the intervention occurs for the last 10 to 20 minutes of their child’s individual sessions. This treatment program was carried out with four children (aged 12 to 14 years) with ASD with a co-current anxiety disorder. The Child and Adolescent symptom Inventory-20, a brief parent-report scale, was used to assess anxiety symptoms. The Anxiety Disorders Interview Schedule for Children/Parents, a clinician rating, was used to assess anxiety. The Social Responsiveness Scale was a parent-report scale that measures their child’s social disability, and the self-reported Multidimensional Anxiety Scale for Children was completed by the children. All measures were administered at baseline, midpoint, endpoint, and 6 months following treatment. The treatment program was effective in reducing anxiety in three of the four subjects and improving the social skills in all four subjects.

To our knowledge, the only detailed report on the efficacy of CBT intervention among adults with ASD comes from Cardaciotto and colleagues [11]. In this study, the subject was a 23-year-old male with AS and co-morbid social anxiety disorder. The intervention included individual CBT over 14 weeks; a clinician who did not administer the CBT examined the effects of the therapy. The subject was assessed at the initial examination (6 months before the intervention), 2 weeks before the intervention, immediately before the initial intervention, during the intervention, immediately after the intervention, and 2 months after the intervention, using the Social Phobia and Anxiety Inventory (SPAI), Liebowitz Social Anxiety Scale (LSAS), and Beck Depression Inventory II. The subject showed improvements across all three measures.

### Objectives

The purpose of this study is to investigate the efficacy of group-based CBT for adults with ASD. Our primary hypothesis is that, through group-based CBT focusing on emotion-regulation and psychoeducation about ASD, adults with ASD can understand their own and others’ emotions and thoughts, exercise emotion-regulation, and increase their knowledge of ASD and self-awareness, especially of their own strengths and weaknesses related to ASD. A small-group adult CBT study protocol will be prepared with reference to previous CBT studies. As noted above, parent training, as well as other forms of intervention, can be carried out along with a child’s CBT. Thus, parents gain a greater understanding of ASD and the necessary modifications to a child’s environment, which is highly effective in enabling a parent being able to adapt to a child with ASD. However, unlike in children, adults with ASD need to understand more about their own strengths and weaknesses, as it is more desirable and practical that they are able to modify their own environment instead of his/her parents. Therefore, this study, which attempts to help improve social adaptation among adults with ASD, comprises two programs: (1) increasing the individual’s emotional awareness and allowing them to acquire appropriate coping skills, and (2) increasing self-awareness through ASD psychoeducation, by learning about the symptoms and biological cause of ASD and the individual’s strengths and weakness associated with it. These programs will be provided regularly, and the effects of these programs will be assessed.

## Methods/Design

### Trial design

**Figure 1:**
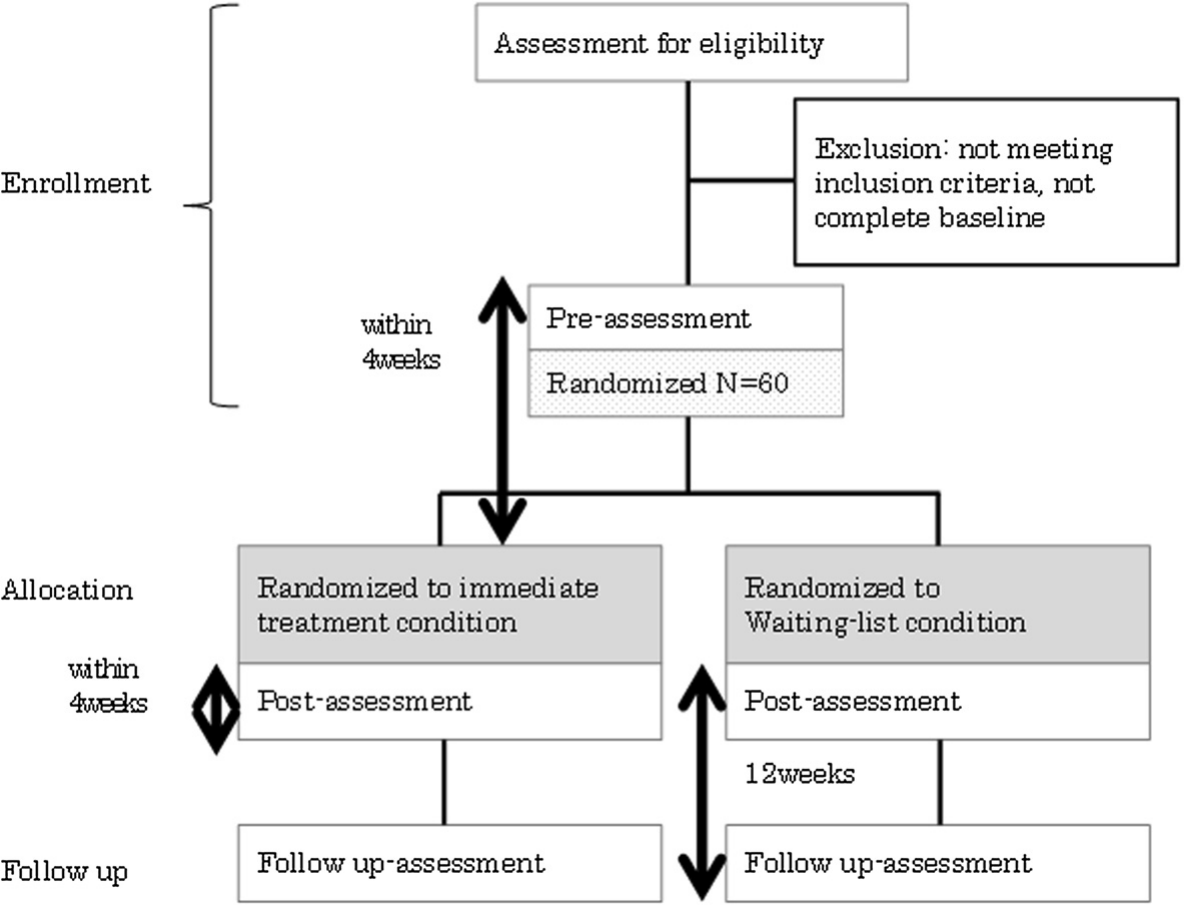
**CONSORT flow chart of the clinical trial.** The allocation of participants will be equal (1:1) across a cognitive-behavioral therapy group and a waitlist control group. All assessment will be administrated at the enrolment, post-intervention, and at 12 weeks follow-up. Both pre- and post-assessment will be performed at 4 weeks before or after the treatment.

This study is a randomized controlled trial. It follows a waitlist control, single-blinded (participants and psychologists who conduct the group-based CBT are not blinded and the assessors of all measures are blinded) design. The allocation of participants will be equal (1:1) across a CBT group (intervention group) and a waitlist control group. All assessment will be administrated by the blinded assessors at the enrolment, post-intervention, and at 12 weeks follow-up. The entire trial design is illustrated in Figure 1. First, an assessment for eligibility will be performed. For individuals who meet the inclusion criteria, a pre-assessment will be performed no more than 4 weeks before the treatment. After pre-assessment, participants will be allocated into the immediate treatment condition or the waitlist condition. After the completion of treatment, post-assessment will be performed within 4 weeks. After an additional 12 weeks, a follow-up assessment will be performed. The pre-assessment and intervention will be conducted at the Department of Child and Adolescent Mental Health, the National Institute of Mental Health, National Center of Neurology and Psychiatry, and the Department of Child Psychiatry, the University of Tokyo Hospital. The allocation and post- and follow-up assessments will be conducted at the Department of Child Psychiatry, the University of Tokyo Hospital.

The target sample size is 60, and registration began on September 1, 2011. This trial was registered in the University Hospital Medical Information Network Clinical Trials Registry and approved by the International Committee of Medical Journal Editors (No. UMIN000006236).

### Ethical consideration

The Ethics Committee of the National Institute of Neurology and Psychiatry (No. A2010-022) and Graduate School of Medicine and Faculty of Medicine at the University of Tokyo have approved the study protocol (No. 2702). All participants will be asked to sign a written informed consent, as approved by the ethical committee of each site, according to the Declaration of Helsinki after receiving a complete explanation of the trial.

### Participants

The participants will be individuals diagnosed with pervasive developmental disorder based on the text revision of the diagnostic and statistical manual of mental disorders, fourth edition (DSM-IV-TR) criteria [18]. In our study, the Cat-kit [16], which was used in the studies of Sofronoff and colleagues, will be used, along with the procedure of those studies. Sofronoff and colleagues had 23 to 25 participants in each of their intervention groups. Therefore we decided to use 25 participants each for the intervention and control groups, recruiting 30 individuals per group to account for potential dropouts.

The inclusion criteria are as follows: (1) age between 18 and 50 years; (2) a full intelligence quotient (IQ) of at least 85 and a verbal IQ of 100 or above (IQ will be evaluated using the Wechsler Adult Intelligence Scale, Third Edition [19]); (3) a specific diagnosis based on the Autism Diagnostic Schedule (ADOS) [20] or the Autism Diagnostic Interview, Revised (ADI-R) [21] with a score above the ASD cut-off; (4) high school graduate or above; (5) knowledge of one’s diagnosis, (6) realization of one’s poor emotional self-awareness/ability to express emotion and poor understanding of others’ emotions and thoughts, and (7) willingness to participate. Individuals with a comorbid psychiatric and/or unstable condition will be excluded (Table 1). The Mini-International Neuropsychiatric Interview [22] will be used to evaluate psychiatric co-morbidity. Any individual who fails to attend more than three sessions will be regarded as 'dropping out', but supplementary instruction will be regarded as equivalent to attendance and will be offered up to three times. Current medication doses should not be increased greatly during the trial. Furthermore, current individual psychological therapy and regular medical treatment will be continued during the intervention. A detailed ASD assessment and diagnosis will be carried out for all intervention candidates. Recruitment of individuals with an ASD diagnosis will be conducted through the Department of Child Psychiatry or Neuropsychiatry at the University of Tokyo Hospital or an advertisement on the University of Tokyo Hospital web site.

**Table 1:** All measures assessed at enrollment, pre-intervention, post-intervention, and follow-up, including primary and secondary outcomes

**Measures**	**Time required (minutes)**	**Dx data**	**Pre-intervention**	**Post-intervention**	**Follow-up**
DSM-IV-TR		○			
M.I.N.I	30		○		
WAIS-III	60	○			
AQ	10	○			
E-SQ	15	○			
ADI-R	(120: parent)	○			
ADOS	90	○			
SRS-A	15	○			
SCQ	(20: parent)	○			
MPMR	15		○	○	○
TAS-20	5		○	○	○
CISS	10		○	○	○
ASD	5		○	○	○
Questionnaire					
WHO-QOL26	10		○	○	○
GAF	30		○	○	○
STAI	10		○	○	○
SPAI	25		○	○	○
LSAS	10		○	○	○
CES-D	5		○	○	○

### Inclusion criteria

•Aged 18–50 years

•Primary diagnosis of autism spectrum disorders (based on criteria by psychiatrists from the text revision of the diagnostic and statistical manual of mental disorders, fourth edition)

•A full intelligence quotient of at least 85 and a verbal intelligence quotient of 100 or above

•Autism Diagnostic Schedule or Autism Diagnostic Interview, Revised score above the autism spectrum disorders cut-off point

•Educational qualifications: high school graduate or beyond

•Informed of his or her diagnosis

•Aware that he or she has poor emotional self-awareness or ability to express emotion and difficulty understanding others’ emotions and thoughts

•Willing to participate in the study

### Exclusion criteria

•A psychiatric comorbid and/or unstable condition

As shown in Table 1, candidates will be provided with a detailed diagnostic confirmation using the Autism-Spectrum Quotient, Japanese version [23], which is a self-report questionnaire measuring the degree to which any adult with a normal IQ possesses traits related to the autistic spectrum. Additional measures will include the Social Responsiveness Scale for Adults, Japanese version [24], which measures the severity of autism spectrum symptoms (completed by a relative), and the Empathizing- Systemizing Quotient, Japanese version [25], which assesses a person’s strength of interest in empathy (defined as the drive to identify with a person’s thoughts and feelings and respond with an appropriate emotion). A person’s strength of interest in systems is defined as the drive to analyze or construct a system. Additionally, interviews using the ADOS for individuals with ASD and interviews with parents using the ADI-R will be conducted. Final participation will be decided after confirmation that the subject meets participation criteria.

ADI-R, Autism Diagnostic Interview, Revised; ADOS, Autism Diagnostic Schedule; ASD, autism spectrum disorders; AQ, Autism-Spectrum Quotient; CES-D, Center for Epidemiological Studies Depression Scale; CISS, Coping Inventory for Stressful Situations; DSM-IV-TR, text revision of the diagnostic and statistical manual of mental disorders, fourth edition; Dx, Diagnosis; E-SQ, Empathizing-Systemizing Quotient; GAF, Global Assessment of Functioning; LSAS, Liebowitz Social Anxiety Scale; M.I.N.I., Mini-International Neuropsychiatric Interview; MPMR, Motion Picture Mind-Reading task; SCQ, Social Communication Questionnaire; SPAI, Social Phobia and Anxiety Inventory; SRS-A, Social responsiveness scale for adults; STAI, State-trait Anxiety Inventory; TAS-20, 20-item Toronto Alexithymia Scale; WAIS-III, Wechsler Adult Intelligence Scale- Third Edition; WHO-QOL 26, World Health Organization Quality of Life 26-item version.

### Assessments/measures

#### Primary outcomes

Our hypothesis is that CBT will help adults with ASD to understand their own and others’ emotions and thoughts and to exercise emotion regulation, by increasing their knowledge of ASD and self-awareness, especially their own strength and weakness related to ASD. Therefore, primary outcome measures will be the 20-item Toronto Alexithymia Scale, Japanese version (TAS-20) [26] scores at post-intervention to evaluate the ability to understand one’s own mind and the percentage of correct response on the Motion Picture Mind-Reading task (MPMR) [27] at post-intervention to evaluate the ability of understanding the minds of others. As other primary outcome measures, we will also adopt the Coping Inventory for Stressful Situations, Japanese version (CISS) [28] scores at post-intervention to assess coping skills during stressful situations and the ASD questionnaire scores at post-intervention to assess the knowledge about ASD and the attitude for ASD. These measures are described in greater detail below.

The TAS-20 is one of the most commonly used measures of alexithymia. Alexithymia is characterized by a difficulty in identifying and describing emotions and the tendency to minimize emotional experience and focus attention externally. This measure is a self-report one and consists of 20 items and three factors: difficulty in identifying feeling, difficulty in describing feeling, externally oriented thinking. Each items is rated from 1 (strongly disagree) to 5 (strongly agree) and the total score ranges from 20 to 100. The time required for this test is about 5 minutes.

The MPMR, developed by Wakabayashi and Katsumata [27], involves advanced theory of mind tasks. Tasks are based on the scenes from a television drama. A total of 41 video clips (each 3 to 11 seconds in length) are included from the television drama series, *Shiroi Kyotō* [The White Tower], which depicts malpractice in a famous Japanese medical school. The participant is asked to judge whether the word or phrase presented on the screen aptly describes the person in each scene. The time required for this test is about 15 minutes.

The CISS determines the preferred coping style of an individual and assesses the relationship between the individual’s coping style and his or her personality. Its results are useful for treatment and intervention planning. The CISS measures three types of coping styles: task-oriented, emotion-oriented, and avoidance coping. This measure is also based on self-reports. The CISS consists of 48 items and each item is rated from 1 (not at all) to 5 (very much). The total score of each of the three coping styles ranges from 16 to 80. The time required for this test is about 10 minutes.

The ASD questionnaire that assesses the knowledge about ASD and the attitude to ASD was developed for this study. The questionnaire involved 10 knowledge-based questions (1 = true to 3 = not true) and five attitude-based questions (1 = disagree to 5 = agree) regarding ASD. The time required for this test is about 5 minutes.

#### Secondary outcomes

We anticipate that they will experience improvement in anxiety and depressive symptoms and their adaptation to their lives as a result of their improved awareness of their own and others’ mind, increased knowledge about ASD, and enhanced coping skills for emotion-regulation. Thus, secondary outcome measures will be the scores of the TAS-20, the CISS and the ASD questionnaire and the percentage of correct response on the MPMR at 12 weeks follow-up and the scores of the State-trait Anxiety Inventory (STAI) [29], the LSAS [30], the SPAI [31], the Center for Epidemiological Studies Depression Scale (CES-D) [32], the Global Assessment of Functioning, Japanese version (GAF) [18] and the World Health Organization Quality of Life 26-item version, Japanese version (WHO-QOL 26) [33] at post-intervention and 12 weeks follow-up. These measures are described in greater detail below.

The STAI is a self-report questionnaire that includes separate measures for state and trait of anxiety. The STAI consists of 20 items each for state and trait of anxiety. Each item for state anxiety is rated from 1 (not at all) to 4 (very much so) and each item for trait anxiety is rated from 1 (almost never) to 4 (almost always). The total score for each ranges from 20 to 80 The time required for this test is about 10 minutes.

The SPAI is a self-report questionnaire that assesses specific somatic symptoms, cognitions, and behaviors across a wide range of potentially fear-inducing situations to measure social anxiety and fear. The SPAI consists of 109 items and two domains: social phobia and agoraphobia. Each item is rated from 0 (never) to 6 (always). The social phobia score ranges from 0 to 192 and the agoraphobia score ranges from 0 to 78. The time required for this test is about 25 minutes.

The LSAS is a questionnaire designed to assess the range of social interactions and performance situations that individuals with social phobia may fear and/or avoid. This measure was designed as a self-report questionnaire, but we use it here in the form of an interview. The LSAS comprises 24 social situations that are each rated for level of fear (0 = none to 3 = severe) and avoidance (0 = none to 3 = usually). The total score ranges from 0 to 144. The time required for the interview is about 10 minutes.

The CES-D is a self-report screening tool for depression and consists of 20 items. Each item is rated from 1 (absent) to 4 (five or more times a week), and the total score ranges from 0 to 60. The time required for this test is about 5 minutes.

The GAF is used by clinicians to make a global assessment of an individual’s adaptive level of functioning on a scale from 0 (poor) to 100 (good). The time required for this interview is about 30 minutes.

The WHO-QOL 26 is used to measure an individual’s subjective sense of wellbeing and quality of life, rather than determining the possible presence of an illness. The WHO-QOL 26 consists of 26 items and four domains: physical health, psychological health, social relationships, and environment. Each item is rated from 1 (poor) to 5 (good) and presented as an average score. The time required for this test is about 5 minutes.

It takes about 1 hour for the participant to fill out all of the questionnaires; therefore, they will be sent via mail to their home 7 to 10 days before the assessment date with careful consideration of the participants’ burden. During the pre-, post-, and follow-up assessments, the GAF and LSAS interviews will take about 30 minutes. The theory of mind tasks, MPMR (done on a PC) will take about 15 minutes.

### Details of the intervention program

The CBT group will receive group therapy over an 8 week period (1 session/week) with each session lasting approximately 100 minutes. Each session will include a short period of relaxation between topics (Table 2). Group therapy consists of four to five adults with ASD and two therapists. The therapist who conducts the group therapy as the leader is the certified developmental psychologist who has a PhD and over 10 years experience working with individuals with ASD. The other therapist, the sub-leader, is also a psychologist and has a Masters degree. One or two typical-development volunteers will also join the group and do the same program as the participants with ASD.

**Table 2:** Timetable of one session

**Schedule**		
5 minutes	Greeting	
30 minutes	Psychoeducation on autism spectrum disorders	
10 minutes	Relaxation	
40 minutes	Work and discussion	
	<Topics>	
	Session 1 Autism spectrum disorders	Session 2 Relaxation
	Session 3 Happiness	Session 4 Comfort
	Session 5 Affection	Session 6 Anxiety
	Session 7 Anger	Session 8 Coping skills
5 minutes	Relaxation	

The program has two parts; one is the psychoeducation on ASD and the other is the emotion-regulation program. Materials for the psychoeducation on ASD prepared for this study will be used for learning and understanding the nature of ASD. The Cat-kit [16] will be used for the emotion-regulation program. The titles of each session are as follows: (1) the characteristics of autism; (2) relaxation 1 and happiness; (3) relaxation 2 and comfort; (4) differences from others and sadness; (5) strengths and anxiety; (6) weaknesses and coping with anxiety; (7) anger and coping methods; (8) summary: autism characteristics and conveying emotions.

During each session, participants will be asked to do some written work. For the part of psychoeducation on ASD, they will describe and present their own preferences, strengths, weaknesses, *et cetera*. In the emotion-regulation program, they will present their experience and physiological changes associated with emotion. The typical-development volunteers will also perform the same tasks. Finally, after completion of the intervention, the individuals with ASD will make out an original notebook containing descriptions of the nature of ASD and emotion-regulation learning and they will be encouraged to use this for continued study.

### Randomization

Enrollment and random allocation will be performed through central registration at the University Hospital Clinical Trial Alliance Clinical Research Supporting System (UHCT ACReSS) at the University of Tokyo. A minimization method will be used with sex as the allocation factor. A third party, who is not involved in this trial, will enroll participants after examining their eligibility and informed consent. Owing to allocation concealment, the random allocation sequence will be provided by UHCT ACReSS and will not be revealed to any researchers or staff until the end of the enrollment period. As this is a single-blinded trial, all assessments will be conducted by raters without knowledge of whether the participant is in the CBT or waitlist conditions.

### Statistical methods

All analyses will be performed using SPSS 20 J (SPSS Inc., Chicago, IL, USA). All data will be analyzed under the intent-to-treat principle. For the primary outcomes, independent *t*-tests will be used to compare changes in scores between the pre-assessment and post-assessment periods between the CBT group and the waitlist control group. The primary outcomes will be analyzed controlling for potential confounds (for example, age, gender, IQ, and clinical characteristics) using regression models. Secondary outcomes will be analyzed using relevant tests at each assessment, controlling for possible confounds as described above. Subgroup analyses will be performed for any possible confounds to differentiate the efficacy of CBT at follow-up.

## Discussion

Expected results are that adults with ASD will be able to identify their own and understand others’ mental states. We further predict that our CBT group will improve their coping skills. Furthermore, secondary symptoms, such as anxiety or depression, should reduce and adaptive behaviors should improve. The novelty of this trial lies in the utilization of CBT for improving emotion regulation among adults with ASD, in contrast with previous studies, which have included children and adolescents (up to age 16) as participants. Previous studies have also focused on the management of anxiety or anger whereas one of our study objectives is to manage the self-regulation of emotion in general. Moreover, previous studies have implemented CBT for children with ASD in addition to parental training. The current study will focus on psychoeducation regarding ASD for adult patients; that is, the current study does not include parent training as a means of the subject’s understanding ASD. Thus, our design should help to determine the efficacy of CBT for adults with ASD. This CBT intervention is the first step in understanding ASD and emotion-regulation in adulthood, especially for persons diagnosed with ASD in adulthood. Our results will hopefully provide promising avenues for developing services for adults with high-functioning ASD in Japan.

### Trial status

At the time of submission, 88% of the participants have been included in the trial. Of these participants, 68% have been tested at follow-up.

## Abbreviations

ADOS: Autism Diagnostic Schedule; ADI-R: Autism Diagnostic Interview, Revised; AS: Asperger’s syndrome; ASD: autism spectrum disorders; Cat-kit: Cognitive Affective Training Kit; CBT: cognitive-behavioral therapy; CES-D: Center for Epidemiological Studies Depression Scale; CISS: Coping Inventory for Stressful Situations; DSM-IV-TR: text revision of the diagnostic and statistical manual of mental disorders, fourth edition; GAF: Global Assessment of Functioning; IQ: intelligence quotient; LSAS: Liebowitz Social Anxiety Scale; MPMR: Motion Picture Mind-Reading task; STAI: State-trait Anxiety Inventory; SPAI: Social Phobia and Anxiety Inventory; TAS-20: 20-item Toronto Alexithymia Scale; UHCT ACReSS: University Hospital Clinical Trial Alliance Clinical Research Supporting System; WHO-QOL 26: World Health Organization Quality of Life 26-item version.

## Competing interests

The authors declare that they have no competing interests.

## Authors’ contributions

MK and YK equally contributed to the design and management of this trial and wrote most of the manuscript. HK made substantial contributions to the conception and design of this trial. KY contributed to the development of the CISS-Japanese version. YK and YK are the directors of each site and made substantial contributions to revising the design and management of this trial. All authors have read and approved the final manuscript.
